# The Effect of a Single Hemodialysis Session on Cochlear Function of Patients with End-Stage Renal Disease

**DOI:** 10.3390/audiolres16030071

**Published:** 2026-05-11

**Authors:** Isil Cakmak Karaer, Irem Pembegul

**Affiliations:** 1 Department of Otolaryngology, School of Medicine, Malatya Turgut Ozal University, Battalgazi, 44210 Malatya, Turkey; 2 Department of Nephrology, School of Medicine, Malatya Turgut Ozal University, Battalgazi, 44210 Malatya, Turkey

**Keywords:** cochlear function, DPOAE, hearing, hemodialysis

## Abstract

**Background/Objectives:** To determine the effect of single-dose hemodialysis treatment on the inner ear in patients with normal hearing functions using the Distortion product otoacoustic emissions (DPOAE) test. **Methods:** A total of twenty-four (24) patients with end-stage renal disease were included in the study. For all patients, the DPOAE test was performed immediately before hemodialysis and 3 h after hemodialysis. Both amplitude and signal-to-noise ratio (SNR) values were compared. **Results:** Compared to pre-dialysis values, the DPOAE test showed a statistically significant decrease in both amplitude and SNR values at all frequencies (1000, 2000, 4000 and 6000 Hz) in patients’ post-dialysis measurements. **Conclusions:** A single hemodialysis treatment has been found to have an adverse effect on cochlear function at both low and high frequencies.

## 1. Introduction

Hemodialysis is an extracorporeal renal replacement therapy in which a patient’s blood is circulated through a machine containing a dialyzer (artificial kidney) with a semipermeable membrane. This process removes metabolic waste products (such as urea), excess water, and corrects electrolyte and acid-base imbalances by allowing solutes to diffuse across the membrane from blood to dialysate (dialysis solution) and vice versa, driven by concentration gradients. Fluid removal (ultrafiltration) is achieved by applying a hydrostatic pressure gradient across the membrane, allowing precise control of volume status [1,2,3]. Indications for hemodialysis include chronic kidney disease (end-stage renal disease), severe acute kidney injury with life-threatening electrolyte or acid-base disturbances, and certain intoxications with dialyzable substances [1,4].

Hemodialysis is associated with an increased risk of sensorineural hearing loss (SNHL) in patients with chronic kidney disease (CKD) and end-stage renal disease (ESRD). Large cohort studies demonstrate that patients undergoing hemodialysis have a higher incidence of SNHL compared to non-dialyzed patients with CKD and the general population, with adjusted hazard ratios indicating a substantially elevated risk [5,6]. The hearing loss is predominantly cochlear and neurosensory in nature, often affecting high frequencies, and may be present even early in the course of dialysis [7,8,9]. Longitudinal evidence further suggests that repeated hemodialysis sessions may lead to progressive cochlear changes, with studies such as that by Saha et al. [10] demonstrating a significant decline in DPOAE amplitudes over successive dialysis sessions.

Distortion product otoacoustic emissions (DPOAEs) are sensitive, noninvasive measures of cochlear (outer hair cell) function and are used to monitor cochlear health in various clinical and research settings [11]. DPOAEs are acoustic signals generated by the cochlea in response to two primary tones (f_1_ and f_2_, with f_1_) [12]. The signal-to-noise ratio (SNR) in DPOAE measurements is a critical parameter that determines the reliability and interpretability of the emission. High SNR is essential for accurate detection and quantification of DPOAEs, especially when constructing input/output (I/O) functions to probe cochlear nonlinearity and compression. Low SNR, particularly at low stimulus levels, can compromise the repeatability and robustness of DPOAE-based assessments [13,14]. A recent study demonstrates that targeting fine-structure peaks in the DPOAE response where SNR is maximized (DPOAE amplitude level) improves the reliability of cochlear compression estimates and the repeatability of I/O function measurements. This approach yields higher Distortion product amplitudes and more consistent results compared to conventional frequency selection, supporting its use in both research and clinical settings [13]. Optimizing stimulus parameters (primary tone levels and frequency ratios) further enhances SNR and the diagnostic utility of DPOAEs [14].

The aim of this study is to evaluate the effect of single-dose hemodialysis treatment on the inner ear in patients with normal hearing functions using the DPOAE test.

## 2. Materials and Methods

A prospective cross-sectional study was conducted in the Department of Otolaryngology at Malatya State Hospital. This study was approved by the Malatya Ethical Review Board (number 2021-61), and it was conducted in accordance with the ethical standards of Declaration of Helsinki. Informed written consent was obtained from the patients.

### 2.1. Study Participants

Twenty-four (24) patients with ESRD were enrolled in the study. Of the 24 patients, 10 (42%) were women and 14 (58%) were men. The median age of patients was 43 (36–55) years.

Patients with ESRD, defined by a GFR persistently below 15 mL/min/1.73 m^2^, were included in the study [15]. To ensure the reliability of the results, all patients underwent otoscopic examination before audiological testing. Only patients with a clean external ear canal and a normal eardrum were included in the study. None of the patients complained of hearing loss, and all patients had normal pure tone audiogram (PTA) tests. Any patients with a family history of hearing impairment or exposure to noise, any neurological disease or systemic illness that could affect hearing, and those who had previously used ototoxic medications, smoked, or consumed alcohol were excluded from the study.

Only patients undergoing their first or second hemodialysis session were included in the study. Patients with a history of hemodialysis more than three times were excluded from the study. This eliminated the adverse effect of chronic hemodialysis treatment on inner ear function.

### 2.2. Otoacustic Emission Test

DPOAE testing was performed on patients one hour prior to hemodialysis. The testing was repeated 3 h after hemodialysis treatment. Before and after hemodialysis, DPOAE test results were compared.

The sound stimulus for the DPOAE consisted of two simultaneous permanent pure tones at different frequencies. Stimulus parameters, L1 = 65, L2 = 55 dB SPL with an f1/f2 ratio of 1.22, were used, and the amplitude of the DPOAE signal was recorded. DPOAE were performed at 1, 2, 4, and 6 kHz frequencies using Otodynamics ILO 288 Echoport equipment (Otodynamics Ltd., London, UK). The parameters of reproducibility percentage, the response (emission strength) value (dB), the Distortion product amplitude and the SNR were evaluated for DPOAE. The averages and standard deviations (SD) of the DPOAE results were calculated.

### 2.3. Statistical Analysis

All analyses were conducted using SPSS 15.0 (SPSS^®^ for Windows 15.0, Chicago, IL, USA). The Shapiro–Wilk test was used to evaluate normality. As the majority of variables demonstrated a non-normal distribution, non-parametric statistics were utilized for the analysis. The Wilcoxon signed-rank test was used to compare pre- and post-dialysis measurements. Results are expressed as median and interquartile range (25th–75th percentiles). A *p*-value < 0.05 was considered statistically significant.

## 3. Results

A single hemodialysis session resulted in a consistent and statistically significant decline in both amplitudes and SNR values across all tested frequencies, indicating a measurable deterioration in outer hair cell function immediately following dialysis.

**Figure 1:**
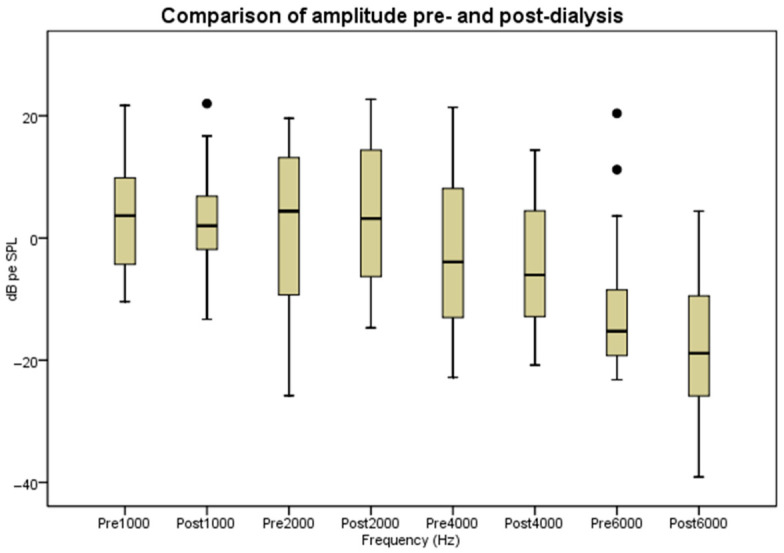
Comparison of amplitude pre- and post-dialysis. dB pe SPL: peak-equivalent Sound Pressure Level.

Post-dialysis amplitudes were reduced at all frequencies when compared with pre-dialysis measurements. As illustrated in Table 1 and Figure 1, these reductions reached statistical significance at 1000 Hz (*p* < 0.001, Z: −3.8), 2000 Hz (*p* = 0.04, Z: −2.1), 4000 Hz (*p* = 0.002, Z= −3.1) and 6000 Hz (*p* = 0.007, Z= −2.7).

**Figure 2:**
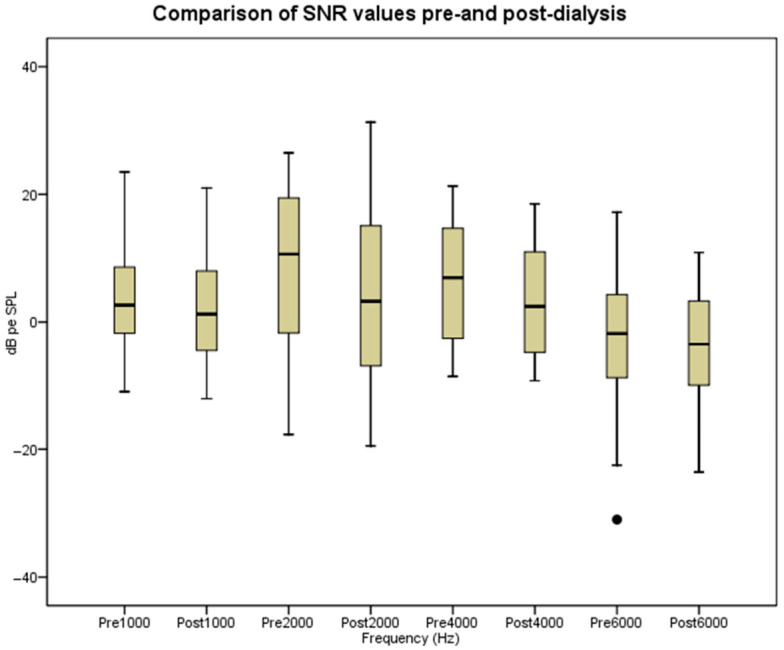
Comparison of SNR values pre-and post-dialysis. SNR: signal-to-noise ratio; dB pe SPL: peak-equivalent Sound Pressure Level.

Similarly, SNR values demonstrated a significant post-dialysis decline. As shown in Table 2 and Figure 2, statistically significant differences were identified at all evaluated frequencies, including 1000 Hz (*p* = 0.002, Z: −2.2), 2000 Hz (*p* = 0.008, Z = −2.6), 4000 Hz (*p* < 0.001, Z = −3.9), and 6000 Hz (*p* = 0.01, Z: −2.5). This parallel reduction in SNR further supports the impact of hemodialysis on cochlear outer hair cell activity.

Despite these measurable audiological changes, no otological symptoms or complications were observed in any patient following a single hemodialysis session.

## 4. Discussion

The present study showed a statistically significant reduction in DP amplitudes and SNR values following a single hemodialysis session. These findings suggest that acute changes in cochlear function may occur in the early post-dialysis period, as reflected by DPOAE measurements obtained at the third hour after dialysis. However, these results should be interpreted with caution given the methodological limitations of the study.

Previous studies have reported an increased prevalence of cochlear dysfunction and sensorineural hearing loss in patients with CKD, particularly among those undergoing long-term hemodialysis [16,17,18]. The mechanisms proposed include metabolic disturbances, vascular changes, and biochemical factors associated with uremia and dialysis treatment [19,20,21]. Nevertheless, the extent to which a single hemodialysis session contributes to acute cochlear dysfunction remains uncertain.

In contrast to the findings of this study, some studies have reported no significant short-term changes in hearing thresholds following a single hemodialysis session, particularly when assessed using PTA [21]. Similarly, Ozturan et al. [22] found no significant differences in PTA or DPOAE parameters between dialysis patients and healthy controls. These discrepancies may be related to differences in study design, sample size, timing of measurements, and the sensitivity of the assessment tools used.

DPOAE testing is considered a sensitive method for detecting outer hair cell dysfunction and may identify subclinical cochlear changes not captured by conventional audiometric methods. In the present study, although no clinically overt hearing loss was detected, significant reductions in DPOAE parameters were observed. This may indicate the presence of transient or subclinical alterations in cochlear function; however, the clinical significance of these findings remains unclear.

The underlying mechanisms for the observed changes are likely multifactorial. Hemodialysis is associated with rapid shifts in fluid and electrolyte balance, as well as changes in blood pressure and cochlear perfusion, which may influence inner ear homeostasis. While such physiological changes could plausibly affect cochlear function, the current study design does not allow for direct determination of causality.

Following dialysis sessions, acute changes in hearing thresholds, particularly at low frequencies, may temporarily occur; this is likely due to rapid changes in endolymph composition. DPOAEs provide a sensitive and objective measure of cochlear function capable of detecting subclinical changes during dialysis [16,17,18]. It has been shown that DPOAEs detect cochlear dysfunction associated with interruptions in cochlear blood flow. Measurable changes in DPOAE levels appear 15–30 s after circulation stops, and phase changes can be detected even earlier (1–5 s) [16]. Such sensitivity may suggest that DPOAEs could be beneficial for real-time monitoring of cochlear function during hemodialysis, particularly in patients at risk for otologic complications. Amplitudes and SNR are often reduced in CKD and hemodialysis patients compared to healthy controls, even in the absence of overt hearing loss [18,23]. A similarity was observed in this study.

In contrast to previous literature, this study assessed both amplitudes and SNR values before and after hemodialysis. Amplitude reflects cochlear nonlinearity, while adequate SNR is essential for the reliability and interpretability of DPOAE measurements. This dual approach represents a major strength of the study. Several limitations should be acknowledged. The relatively small sample size limits generalizability. Cochlear function was evaluated only at a single post-dialysis time point (three hours), precluding assessment of the persistence or reversibility of the observed changes. Potential confounders, including variability in dialysis parameters, fluid and electrolyte shifts, and patient comorbidities, were not fully controlled. Finally, the observational design precludes causal inference regarding the relationship between hemodialysis and cochlear function.

## 5. Conclusions

In this study, a single hemodialysis session was associated with a measurable decline in cochlear function, as reflected by reduced amplitudes and SNR values across frequencies. These findings suggest a possible acute effect of hemodialysis on outer hair cell function; however, they should be interpreted cautiously due to study limitations. Further large-scale, controlled, and longitudinal studies are needed to confirm these observations and determine their clinical significance.

## Figures and Tables

**Table 1:** Comparison of amplitude pre- and post-dialysis.

Test Frequency (Hz)	Pre-Dialysis			Post-Dialysis			*p*	*Z*
	Median	25th	75th	Median	25th	75th		
1000	3.1	−5.2	8.6	1.8	−3.1	5.1	<0.001	−3.8
2000	4.9	−7.5	12.2	2.6	−8.5	11.9	0.04	−2.1
4000	−4.1	−12.7	7.1	−6.9	−20.3	3.3	0.002	−3.1
6000	−16.8	−24.5	−9.5	−19.5	−24.7	−13.1	0.007	−2.7

Data are presented as median ± IQR. *p* < 0.05 was considered statistically significant.

**Table 2:** Comparison of SNR values pre- and post-dialysis.

Test Frequency (Hz)	Pre-Dialysis			Post-Dialysis			*p*	*Z*
	Median	25th	75th	Median	25th	75th		
1000	4.6	−0.8	9.7	3.5	−3.1	8.9	0.002	−2.2
2000	11.9	2.2	17.1	8.5	0.5	16.5	0.008	−2.6
4000	7.9	−4.1	14.1	4.7	−6.5	10.4	<0.001	−3.9
6000	−1.5	−7.6	3.2	−3.95	−8.4	2.5	0.01	−2.5

SNR: signal-to-noise ratio. Data are presented as median ± IQR. *p* < 0.05 was considered statistically significant.

## Data Availability

The datasets generated and analyzed during the current study are not publicly available due to privacy and ethical restrictions but are available from the first author, Dr. Cakmak Karaer, upon reasonable request.
